# Chloroform and Its Management

**Published:** 1856-04

**Authors:** 


### ARTICLE X.

### *Chloroform and its Management.*

In our last number we made some remarks upon the occasional fatal effects of this anæsthetic, and their probable cause. Since the publication of that article, another interesting discussion of the question has taken place in the Medical Society of London. It is of such general interest that we have concluded to make a brief abstract of the report given in the Medical Times and Gazette of February 2nd.

Dr. Snow read a paper in which he mentioned the results of certain experiments with chloroform upon the lower animals. In them, when the vapor was sufficiently diluted with air, the respiration failed first, and the circulation ceased afterwards, for want of the stimulus of red blood. When, however, the vapor is too concentrated, it is absorbed from the lung, and circulating through the coronary arteries, sets directly upon the heart, stopping its pulsation. In this manner he believes the various accidents to have happened.

He then went on to reply to Dr. Black's theory, that death took place by simple asphyxia, and assigned the following reasons for believing it to be incorrect.

"1. The process of dying by asphyxia, occupies from four to nine minutes, after the access of air to the lungs is completely cut off; but no medical man could overlook the fact, that his patient was not breathing, and persevere in preventing his doing so for this length of time.

"2. In the greater number of cases of death from chloroform, the patient really inhaled the vapor, so as to be quite insensible from its effects, before the symptoms of danger set in. Out of 44 deaths from chloroform, 7 took place when the surgeon was just about to begin the operation; 12 occurred during its performance; in 8 cases the operation, being of short duration, was completed before it was discovered that the patient had expired; and in the remaining 17 cases, the inhalation was discontinued, at some period of its progress, owing to the sudden occurrence of alarming symptoms.

"3. In every case in which the state of the pulse had been noticed at the time of the accident, it was found to cease suddenly and abruptly. This is totally different from what occurred in asphyxia, where the pulse retained its strength for some time, and then gradually diminishes in frequency and force.

"4. In twelve of the cases of death from chloroform, the face was observed to become suddenly pale at the moment when symptoms of danger set in. This symptom was indicative of cardiac syncope, and was incompatible with asphyxia; it had probably occurred in many cases where it was not recorded.

"5. In several of the cases in which death occurred during the performance of the operation, attention was first called to the patient's danger by the sudden stopping of the hemorrhage; a symptom which also proves death by syncope, and not by asphyxia.

"6. When animals are killed suddenly by chloroform, so as to imitate the accidents to the human subject, the blood is found to be of a florid color in the lungs immediately after death.

"7. Except sometimes in the case of children and lunatics, it is not the custom to restrain the patient, so long as he is conscious. If he complain of the frequency of the vapor, it is accommodated to his feelings; and therefore, it is impossible that death should take place from the cause indicated by Dr. Black, unless in children and lunatics, to whom no accident from chloroform have yet happened.

"8. The vapors of sulphuric ether is as pungent as that of chloroform, but accidents do not happen during its use.

"A serious error with regard to chloroform, is to suppose the patient safe so long as he had sufficient air for the purposes of respiration; the truth being, the more air he breathes, the greater is his danger, if the air be too highly charged with vapor. The air breathed by the patient should never contain more than 5 per cent. of chloroform; if it contain 8 or 10 per cent. it is liable to cause sudden death by suspending the action of the heart. He recommended the use of an apparatus, for regulating the quantity of vapor of chloroform in the air; but those who prefer to use a handkerchief or sponge, may avoid risk, by diluting the chloroform with an equal measure of rectified spirit before using it. He considers that artificial respiration, very promptly and efficiently performed, affords the best prospect of success."

In the discussion which followed the reading of this paper, many interesting points were discussed. The success of different physicians in the use of artificial respiration varied very materially. Some had found it of no service, while others had perfectly succeeded.

